# Supplementary material for: Combined FCS and PCH Analysis to Quantify Protein Dimerization in Living Cells
Source: Int J Mol Sci. 2021 Jul 7;22(14):7300. doi: 10.3390/ijms22147300 (PMC8307594; doi:10.3390/ijms22147300)
Supplement: Supplementary file 1 [file ijms-22-07300-s001.zip › ijms-1255310-supplementary.pdf]

International Journal of *Molecular Sciences*

MDPI

Article

Combined FCS and PCH analysis to quantify protein dimerization in living cells

Laura M. Nederveen-Schippers, Pragya Pathak, Ineke Keizer-Gunnink, Adrie H. Westphal, Peter J.M. van Haastert, Jan-Willem Borst, Arjan Kortholt and Victor Skakun

Supplementary Information

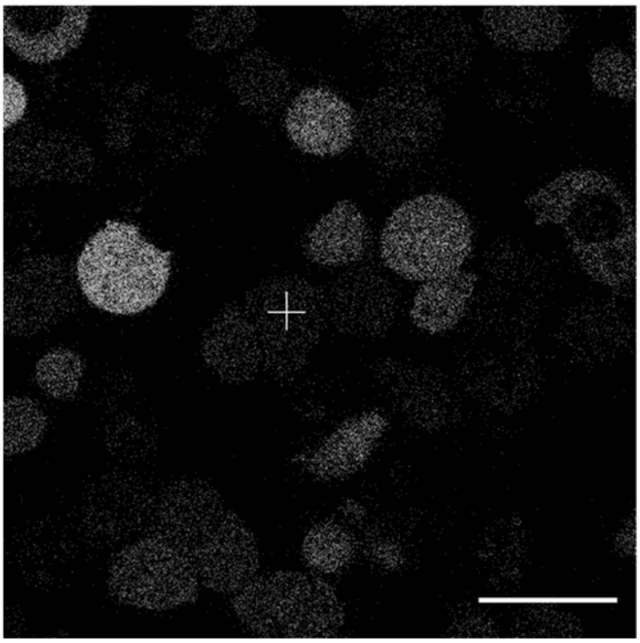

Figure S1. Selection of cells. Green fluorescent images of Dictyostelium cells during the FCS experiment. Shown are vegetative GFP-expressing Dictyostelium cells in the presence of 2% DMSO. The crosshair (+) was placed on relatively dim cells, for a better resolution. Scale bar: 20 μm.

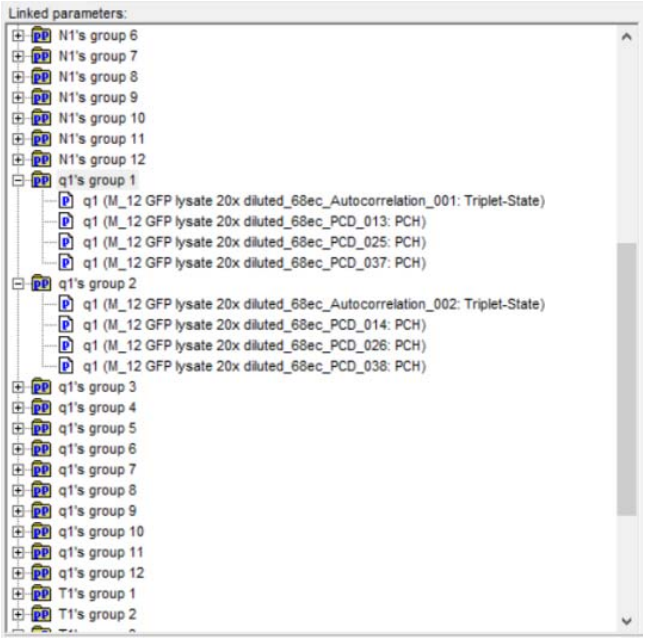

Figure S2. Grouping of parameters per trace. Illustration of linking parameter q1 per trace. Three PCD curves and one ACF curve derived from the same 5-second trace were grouped. In this example twelve groups were formed for parameter q1 and twelve groups for parameter N1.

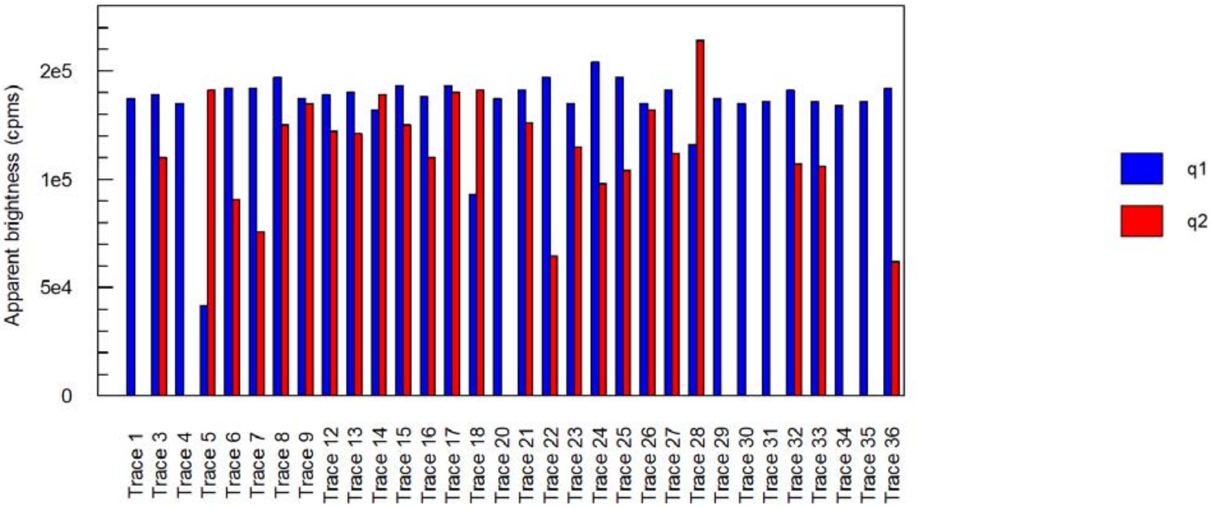

Figure S3. Brightness, estimated in 2 components fit when all parameters are free. Representative example of the initial 2 component global analysis of monomer-dimer mixtures (50%) with free parameters. F_{trip}, τ_{trip} and τ_{diff} were grouped in the global analysis of only ACFs, and their outcomes were subsequently fixed in the combined global analysis of ACFs and PCDs, while $\tau_{diff\,2}$, q_1, q_2, N_1 and N_2 were free and only grouped per trace (parameters of one ACF and three PDC curves were grouped per trace, not all traces together). The sample shown here is a mix of 50% GFP and 50% diGFP in cell lysate. The brightness of GFP and diGFP on the same measurement day were 0.92 ± 0.015 and 1.95 ± 0.030 x 10^5 cpms, respectively. Average q_1 = 1.34 ± 0.20 x 10^5 cpms; average q_2 = 1.15 ± 0.25 x 10^5 cpms, which is in-between the brightness GFP and diGFP. For some traces q_2 could not be determined.

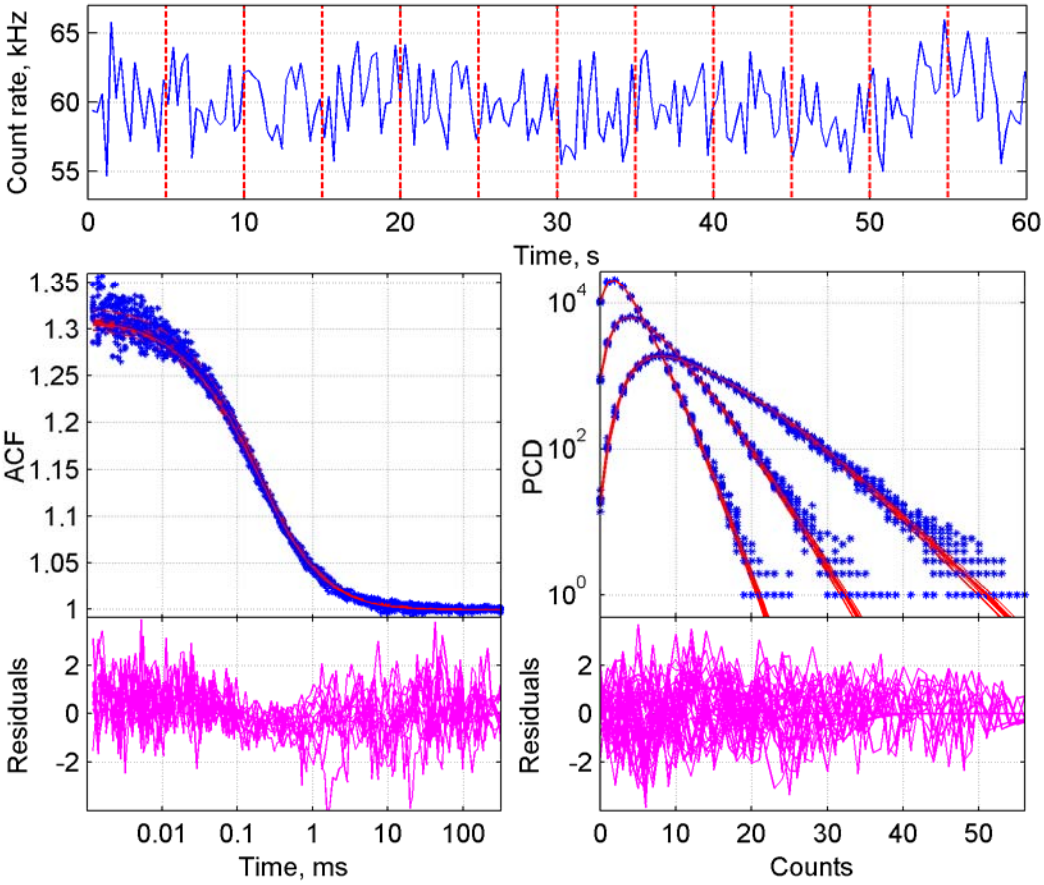

Figure S4. Results of 2-component FSC+PCH global analysis of the ACF and PCD curves from a monomer-dimer equilibrium in cell lysate. The analysis was based on the same data as **Error! Reference source not found.**. Top panel: raw FFS data showing photon counts over time, from which the ACF and PCD curves were calculated. The measurement was divided into 12 5-second traces, as indicated by vertical lines. Bottom left: fit of all ACF curves, with residuals below. Bottom right: fit of all PCD curves, with residuals below. PCD curves were generated with three different time steps of 5 x 10^{-5}, 1 x 10^{-4} and 2 x 10^{-4} s. .

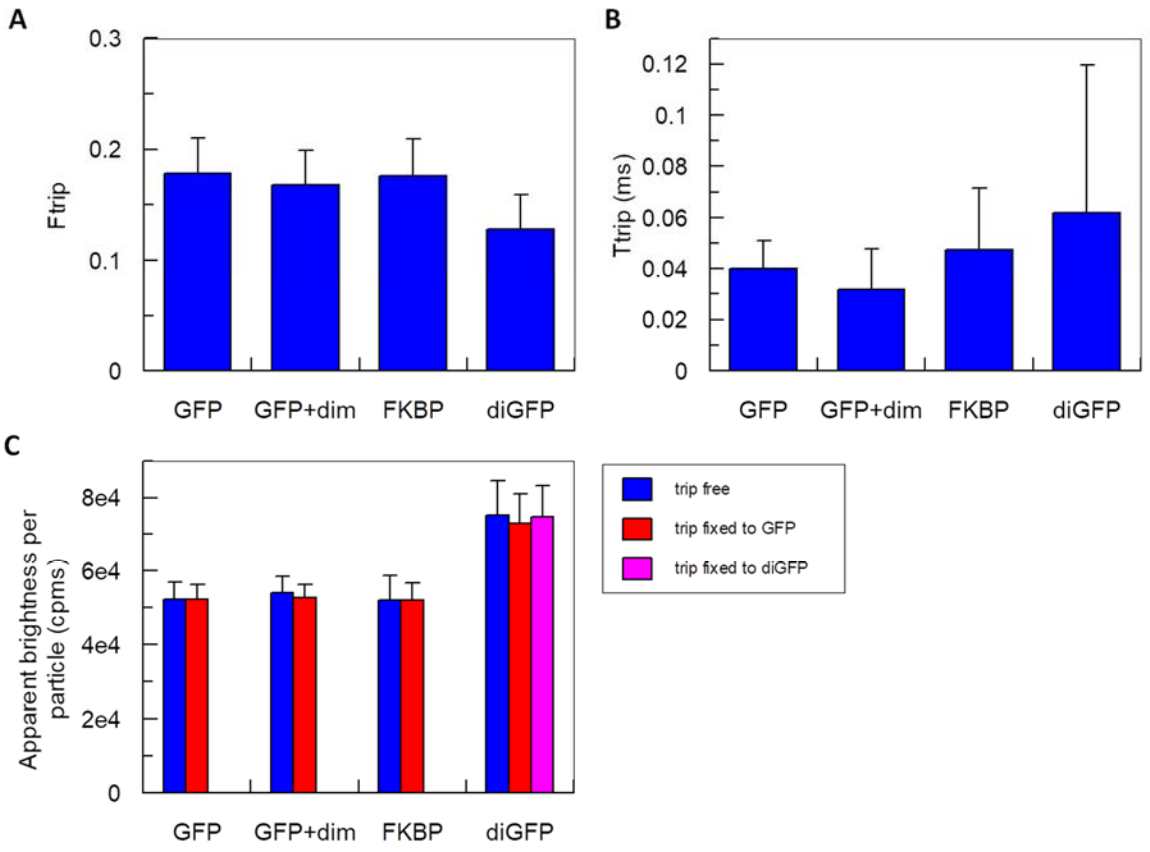

Figure S5: Triplet state parameters may be fixed for all cells. A: Average triplet state fraction (F_{trip}) as determined in the ACF analyses from one measurement day. B: Average triplet state time (τ_{trip}) as determined in the ACF analyses from the same measurement day. C: Average brightness from all cells per sample of one measurement day, with either fixed or free triplet state parameters. Free triplet state parameters were determined per cell in the ACF analysis and subsequently fixed in the combined BDGA analysis. Fixed triplet state parameters were determined by the average of all GFP (or diGFP) cells and fixed in both the ACF and the BDGA analysis. Error bars: standard deviations (SD); 'trip': triplet state parameters.

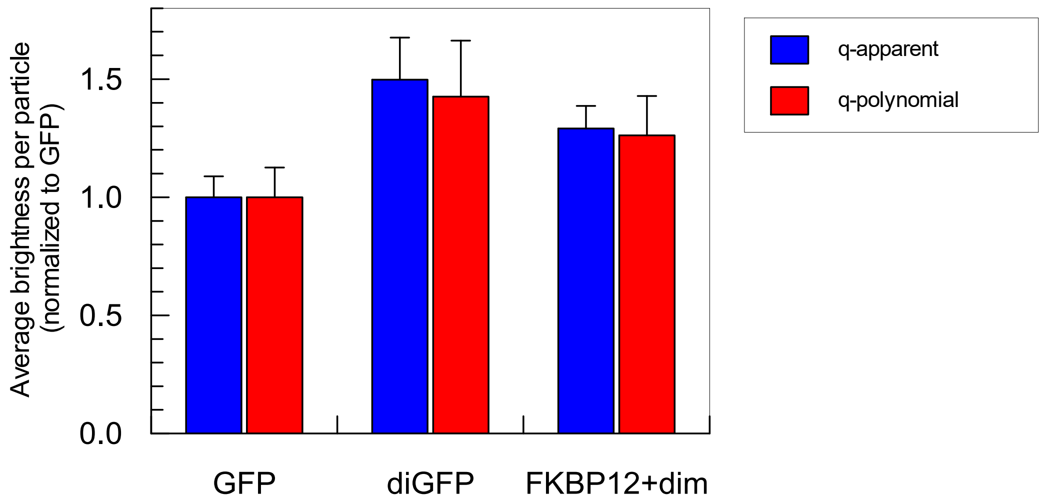

Figure S6. Similar results for the 3D Gaussian and the Polynomial model. Calculations have been repeated with the polynomial model in FFS Data Processor, for a subset of seven cells per sample from one measurement day. q-apparent indicates the apparent relative brightness calculated with the Gaussian model, without correction for F_{C1}. q-polynomial indicates the brightness as calculated with the polynomial model. For both models the same cells have been used for comparison. All brightness values have been normalized to the brightness of GFP control.

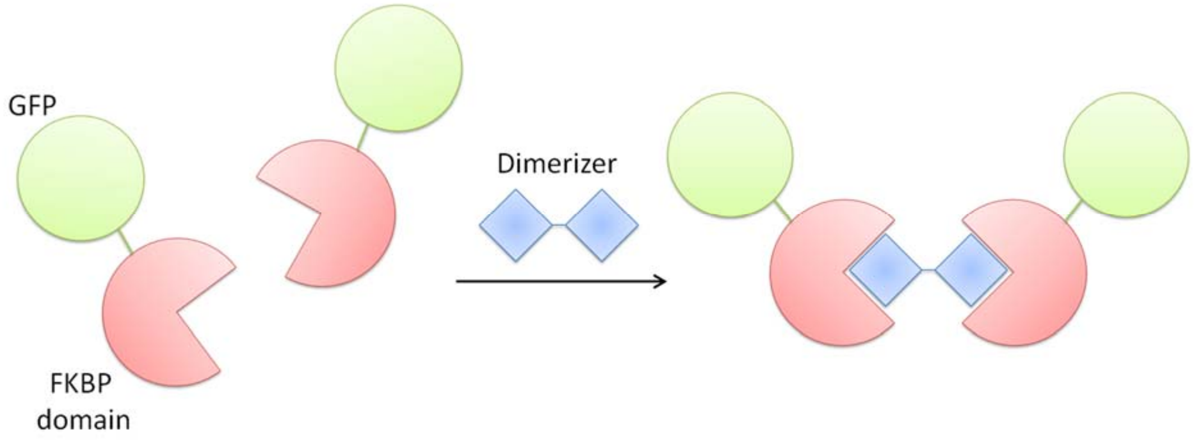

Figure S7. Ligand induced FKBP12 dimerization. Cartoon of the FKBP12 binding domain linked to GFP. When B/B homodimerizer ('Dimerizer') is added to the cells, it symmetrically binds and connects two FKBP12 domains, inducing an indirect link between two GFP fluorophores.

Table S1. List of parameters. The definition and status of the parameters are indicated. All parameters from either one or two component analysis are listed together. 'Fixed to (the value of) GFP' means fixed to the average of the GFP measurements of the same measurement day. Free: parameter was not grouped, nor fixed to a value. Grouped: parameter was grouped between different traces, but not fixed to a value. Grouped per trace: different ACF and PCH curves from one trace were grouped together, but not fixed to a value. Fixed: parameter was fixed to a specific value that has been determined before.

Parameter	Explanation	Status in ACF analysis	Status in BDGA
F_1	Fraction of component 1	Free	-
G_{inf}	Value of ACF curve at infinity	Free	Free
F_{trip}	Triplet state fraction	Fixed to the value of GFP	Fixed to the value of GFP
τ_{trip}	Triplet state relaxation time	Fixed to the value of GFP	Fixed to the value of GFP
τ_{diff} ($\tau_{diff\,1}$)	Residence time of component 1	Grouped	Fixed to the value from the ACF analysis
a	Structural parameter	Fixed to the value of R110	Fixed to the value of R110
N	Number of particles in the confocal volume	Free	-
N_1	Number of particles in the confocal volume (of component 1 or 2)	-	Grouped per trace
Fc_1	Out-of-focus correction factor	-	Grouped
τ_{dt}	Dead-time correction	-	Grouped
Bg	Background	-	Fixed to 0
q_1	Molecular brightness	-	Grouped per trace
χ^2	Fit criterion	*Output*	*Output*
**Additional parameters in case of 2-component analysis of lysate data:**			
F_2	Fraction of component 2 (if applicable)	Free	-
N_2	Number of particles in the confocal volume (of component 2)	-	Grouped per trace
$\tau_{diff\,2}$	Residence time of component 2	Fixed to the value of diGFP (and $\tau_{diff\,1}$ fixed to GFP)	Fixed to the value of diGFP (and $\tau_{diff\,1}$ fixed to GFP)
q_2	Brightness parameter	-	Grouped per trace

Table S2. Example of BDGA output in FFS Data Processor. The standardized BDGA method was applied on the measurement of a cell with FKBP12-GFP and dimerizer (the same cell with FKBP12 as in **Error! Reference source not found.** and **Error! Reference source not found.**). Some values have been fixed to the values from GFP, while others have been fixed to the values from the ACF analysis of this particular cell, as summarized in **Error! Reference source not found.**. T1: $\tau_{diff\,1}$.

Name	Value	Minimum	Maximum	Fixed
Fc1's group	0.927	0	3	False
Ftrip's group 10	0.178	0	0.999	True
Ftrip's group 12	0.178	0	0.999	True
Ftrip's group 13	0.178	0	0.999	True
Ftrip's group 14	0.178	0	0.999	True
Ftrip's group 2	0.178	0	0.999	True
Ftrip's group 4	0.178	0	0.999	True
Ftrip's group 5	0.178	0	0.999	True
Ftrip's group 8	0.178	0	0.999	True
Ftrip's group 9	0.178	0	0.999	True
Ginf	1	0.5	1.5	False
Ginf	0.999	0.5	1.5	False
Ginf	1.001	0.5	1.5	False
Ginf	1.003	0.5	1.5	False
Ginf	1.004	0.5	1.5	False
Ginf	1.003	0.5	1.5	False
Ginf	1.001	0.5	1.5	False
Ginf	1.003	0.5	1.5	False
Ginf	1.003	0.5	1.5	False
N1's group 10	20.15	0	100	False
N1's group 12	17.459	0	100	False
N1's group 13	17.81	0	100	False
N1's group 14	16.525	0	100	False
N1's group 2	21.665	0	100	False
N1's group 4	19.628	0	100	False
N1's group 5	20.205	0	100	False
N1's group 8	19.069	0	100	False
N1's group 9	18.775	0	100	False
T1's group 10	1.018	1.00E-03	10	True
T1's group 12	1.018	1.00E-03	10	True
T1's group 13	1.018	1.00E-03	10	True
T1's group 14	1.018	1.00E-03	10	True
T1's group 2	1.018	1.00E-03	10	True
T1's group 4	1.018	1.00E-03	10	True
T1's group 5	1.018	1.00E-03	10	True
T1's group 8	1.018	1.00E-03	10	True
T1's group 9	1.018	1.00E-03	10	True
Tdt's group	6.34E-05	1.00E-06	1.00E-03	False
Ttrip's group	4.00E-02	1.00E-06	1	True
a's group	5.9	1.001	20	True
bg's group	0	0	1.00E+06	True
q1's group 10	6.30E+04	0	1.00E+07	False
q1's group 12	6.15E+04	0	1.00E+07	False
q1's group 13	5.64E+04	0	1.00E+07	False
q1's group 14	5.74E+04	0	1.00E+07	False
q1's group 2	7.50E+04	0	1.00E+07	False
q1's group 4	7.55E+04	0	1.00E+07	False

q1's group 5	7.67E+04	0	1.00E+07	False
q1's group 8	6.94E+04	0	1.00E+07	False
q1's group 9	7.05E+04	0	1.00E+07	False

Table S3. Comparison of various settings of the 2-component analysis of one monomer-dimer mix. All analyses have been performed on the same measurement of one sample, which was a mix of 50% GFP and 50% diGFP, consisting of 12 traces of 5 seconds. 'q and τ free': $\tau_{diff\,1}$ was grouped and calculated in the global analysis of only ACFs and fixed in BDGA, while $\tau_{diff\,2}$, q_1 and q_2 were only grouped per trace not for all traces together. 'q free': $\tau_{diff\,1}$ and $\tau_{diff\,2}$ were fixed to the values found for GFP and diGFP (**Error! Reference source not found.**), while q_1 and q_2 were free. 'r = 1.8' (or 2): $\tau_{diff\,1}$ and q_1 were fixed to the values found for GFP and $\tau_{diff\,2}$ to the value found for diGFP, while q_2 was fixed to 1.8x or 2x the value of q_1. 'q_2 of diGFP': $\tau_{diff\,1}$, $\tau_{diff\,2}$, q_1 and q_2 were fixed to the values found for GFP and diGFP. All parameters except N and $\tau_{diff\,2}$ are presented with confidence intervals, calculated as asymptotic standard errors (ASE), as reported by the software. F_{trip}: triplet state fraction; τ_{trip}: triplet state time; $\tau_{diff\,1}$ and $\tau_{diff\,2}$: diffusion parameters as reported by the software; N_1 and N_2: number of particles in the confocal volume, SD is indicated; q_{1true} and q_{2true}: true brightness, with ASEs representing uncertainties of estimated parameters obtained in analyses performed per one trace. We additionally calculated standard deviations of brightness between traces (SD of q). χ^2: global analysis fit criterion value. Cpms: counts per molecule per second. Fixed values are indicated in italics and have been determined in **Error! Reference source not found.**. Values of N which are closest to the expected 50-50 ratio are indicated in bold.

Analysis method	F_{trip} (x10^{-2} ms)	τ_{trip} (x10^{-2} ms)	$\tau_{diff\,1}$ (ms)	$\tau_{diff\,2}$ (±SD)	N_1 (±SD)	N_2 (±SD)	q_{1true} (x10^4 cpms)	SD q_{1true} (x10^4 cpms)	q_{2true} (x10^4 cpms)	SD q_{2true} (x10^4 cpms)	χ^2
2-comp (q and τ free)	7.13 ± 0.61	1.47 ± 0.25	0.171 ± 0.004	6.2 ± 11.2	1.73 ± 0.90	5.28 ± 3.98	6.58 ± 0.242	2.089	2.65 ± 0.21	2.49	0.971
2-comp (q free)	8.07 ± 0.46	1.91 ± 0.24	*0.151*	*0.221*	1.89 ± 1.37	2.23 ± 1.40	6.08 ± 0.222	3.101	5.42 ± 0.18	2.24	0.832
2-comp (r = 1.8)	8.07 ± 0.46	1.91 ± 0.24	*0.151*	*0.221*	**2.00 ± 0.18**	**1.76 ± 0.12**	*3.20*		*5.76*		1.068
2-comp (r = 2)	8.07 ± 0.46	1.91 ± 0.24	*0.151*	*0.221*	2.65 ± 0.14	1.23 ± 0.08	*3.20*		*6.4*		0.924
2-comp (q_2 of diGFP)	8.07 ± 0.46	1.91 ± 0.24	*0.151*	*0.221*	1.19 ± 0.27	2.49 ± 0.17	*3.20*		*5.12*		1.294

Table S4. Comparison of various q-ratios of multiple monomer-dimer mixes. All samples have been measured on the same day. Fixed values are indicated in italics. Since this analysis was performed in an earlier stage, F_{trip} and τ_{trip} have been determined per sample (not shown), instead of fixed to the same value for all analyses, and $\tau_{diff\,1}$, $\tau_{diff\,2}$ and q_1 have been fixed to different values than determined for GFP and diGFP in **Error! Reference source not found.**. N_1 and N_2 have been calculated into percentages by the formulas $N_1\% = N_1/(N_1 + N_2)$ x100% and N2% = $N_2/(N_1 + N_2)$ x100%. The N_2% closest to the % diGFP (input) are indicated in bold, along with the corresponding q_2/q_1 ratio. The N_2% may become even closer to the diGFP% when $\tau_{diff\,2}$, $\tau_{diff\,2}$ and/or q_1 are varied. As shown here, optimal settings varied a lot per sample and therefore no standardized procedure could be determined.

% diGFP	$\tau_{diff\,1}$	$\tau_{diff\,2}$	q_1	$r = q_2/q_1$	N_1 %	N_2 %
10	*0.14*	*0.2*	*5.50E+04*	*1.8*	93.2	6.8
	0.14	*0.2*	*5.50E+04*	***1.7***	91.8	**8.2**
12.5	*0.14*	*0.2*	*5.50E+04*	***1.8***	88.2	**11.8**
	0.14	*0.2*	*5.50E+04*	*1.7*	85.7	14.3
33.3	*0.14*	*0.2*	*5.50E+04*	*1.8*	71.2	28.8
	0.14	*0.2*	*5.50E+04*	*1.6*	56.2	43.8
	0.14	*0.2*	*5.50E+04*	***1.7***	64.7	**35.3**
50	*0.14*	*0.2*	*5.50E+04*	***1.8***	50.0	**50.0**
67.7	*0.14*	*0.2*	*5.50E+04*	*1.6*	14.0	86.0

	0.14	*0.2*	*5.50E+04*	*1.8*	45.3	54.7
	0.14	*0.2*	*5.50E+04*	***1.7***	31.5	**68.5**
87.5	*0.14*	*0.2*	*5.50E+04*	*1.8*	28.2	71.8
	0.14	*0.2*	*5.50E+04*	*1.6*	7.5	92.5
	0.14	*0.2*	*5.50E+04*	***1.7***	9.7	**90.3**
90	*0.14*	*0.2*	*5.50E+04*	*1.8*	30.6	69.4
	0.14	*0.2*	*5.50E+04*	***1.6***	9.6	**90.4**

Step-by-step protocol of FCS Data analysis using the BDGA methodology

1. *Data preparation*

   a. For cellular data, split each measurement into traces of 3-5 seconds.
      Note. *In vitro* data is more stable and may consist of longer traces.
   b. For each trace, calculate one ACF (time step 1 x 10^{-7} s (cells) or 2 x 10^{-7} s (lysate); point count 140) and three PCDs (point count 32), the latter with different time steps depending on the total fluorescence intensity, as described in section 3.1.1.

2. *Curve fitting with the 1-component BDGA model*

2.1 ACF only

1. Fit globally all ACF traces from one measurement using the free diffusion 3D Gaussian model with the triplet-state term (Eq. 1).

   a. Group parameters F_{trip}, τ_{trip}, and τ_{diff1} between traces, and fix parameter a to the value found for the Rhodamine 110 calibration dye.
   b. Generate initial guesses.
   c. Run the analysis.
   d. Remove traces with a very aberrant fit (e.g. with a steep drop of the ACF curve on the right side of the plot, or an overall alleviation of the curve compared to the other traces; this correlated with irregularities in the original traces), and run the analysis again.
   e. The chi-square value is a measure for how good the fit is and should be close to 1, but any value between 0.7 and 1.4 is considered acceptable.

2. Calculate averages F_{trip} and τ_{trip} from all GFP measurements of one day and then fix them for all samples of that day (except diGFP), as recommended in section 3.2.

Note. In the 2-component BDGA analysis, parameters τ_{diff2}, N_2, and q_2 were added to the model and grouped per trace. F_{trip}, τ_{trip}, τ_{diff1} and a were fixed like in the 1-component model, but τ_{diff2} was not, to allow for differences between traces.

2.2 Combined ACF and PCD

1. Fit globally all ACF and PCD curves from the same measurement using the protocol described in detail in [1]. The FCS and PCH models are calculated as described in section 2.1.

   a. Group the parameters τ_{diff1}, F_{trip}, N_1, and q_1 per trace (one ACF and three PCDs), while grouping τ_{trip}, a and some other parameters (Fc_1, τ_{dt}, bg) for all traces together.
   b. Fix parameters a, F_{trip}, and τ_{trip} to the same values as fixed during the analysis of only the ACF curves, while fix τ_{diff1} to the value found during that ACF analysis.
   c. Run the analysis immediately without generation of initial guesses.
   d. Examine the fits and residuals of the ACF and the PCD curves for aberrations, before exporting the resulting parameter values to a text file.

3. *Postprocessing of fit results*

   a. Import the resulting data in a spreadsheet program like Microsoft Excel.
   b. Calculate the average q_1, q_2, τ_{diff1} and τ_{diff2} per cell.

c. Normalize obtained brightness values towards GFP on that day.
d. Average both brightness and diffusion times between all cells of multiple days.
e. Convert τ_{diff1} and τ_{diff2} into D_1 and D_2 by the formulas given in section 2.1.
f. The significance of apparent differences between samples may be determined using the standard two-sided t-test with unequal variances.
g. Calculate standard deviations from the variation between cells, based on the normalized data from multiple measurement days.

References

1. Skakun, V. V.; Digris, A. V.; Apanasovich, V. V. Global analysis of autocorrelation functions and photon counting distributions in fluorescence fluctuation spectroscopy. *Methods Mol. Biol.* **2014**, *1076*, 719–741, doi:10.1007/978-1-62703-649-8_33.
